# Clinical challenges and advances in HPV-related oropharyngeal squamous cell carcinoma

**DOI:** 10.1097/JS9.0000000000002827

**Published:** 2025-06-30

**Authors:** Yao Shi, Xin Zhao, Yuzhen Wen, Yamin Dong, Yue Wang

**Affiliations:** a Department of Oral and Maxillofacial Surgery, Zibo central hospital, ZiBo, Shandong, China; b Department of stomatology, Zibo central hospital, ZiBo, Shandong, China; c Department of Oral and Maxillofacial Surgery, The Affiliated Hospital of Qingdao University, Qingdao, China; d School of Stomatology, Qingdao University, Qingdao, China; e Key Lab of Oral Clinical Medicine, The Affiliated Hospital of Qingdao University, Qingdao, China

We read with great interest the review article “Recent advances in HPV biotechnology: understanding host-virus interactions and cancer progression—a review.” recently published in the *International Journal of Surgery*^[1]^. The article provides a comprehensive overview of HPV–host interactions, mechanisms of oncogenesis, immune regulation, and therapeutic innovations across a range of HPV-associated malignancies. This work significantly advances our understanding of viral-driven cancers and highlights emerging opportunities for translational research^[2]^.

However, we believe that the discussion on HPV-related oropharyngeal squamous cell carcinoma (HPV + OPSCC), one of the fastest rising subtypes of head and neck cancer, could be further expanded. In recent years, HPV + OPSCC has presented new clinical, biological, and public health challenges worldwide. Based on recent scientific advances and clinical experience, we aim to provide further commentary on the current clinical challenges, pathogenesis, treatment strategies, and prevention efforts for HPV + OPSCC, building upon the excellent foundation laid by the authors.

## Current challenges

OPSCC arises from the mucosal epithelium of the oropharynx, including the tonsils, base of tongue, soft palate, and pharyngeal walls^[3]^. Historically, tobacco and alcohol were recognized as the predominant etiological factors for this malignancy^[3]^. With global declines in tobacco use and alcohol consumption, it was anticipated that OPSCC incidence would similarly decrease. Contrarily, epidemiological trends reveal a steady increase in OPSCC incidence, particularly in high-income countries such as the United States, the United Kingdom, and Northern Europe^[4]^. In the United States, the incidence of HPV + OPSCC now exceeds that of cervical cancer at the population level. This is primarily driven by increasing HPV + OPSCC incidence in men, while the incidence of cervical cancer in women is decreasing due to successful screening and vaccination efforts^[5]^.

**KEY CONTRIBUTIONS AND LEARNING POINTS**

This commentary expands upon the existing literature by providing an updated synthesis of current clinical challenges, recent molecular and immunological insights, evolving treatment paradigms, and emerging prevention strategies specific to human papillomavirus (HPV) + OPSCC—areas that were only briefly addressed in the original review.

Learning points from this commentary include the following:

1. Understanding the epidemiological trends driving the rising global burden of HPV + OPSCC, particularly in male populations.
2. Recognizing diagnostic limitations and the need for improved HPV detection and risk stratification.
3. Appreciating novel molecular mechanisms of immune evasion and emerging targets for immunotherapy.
4. Gaining insight into ongoing clinical trials exploring treatment de-intensification and immunotherapy integration.
5. Highlighting the critical role of HPV vaccination and the promise of circulating tumor HPV DNA (ctHPV DNA) assays for surveillance and early detection.

By summarizing these advances, we aim to complement the original review and provide a practical, clinically relevant perspective to guide future research and patient management in HPV + OPSCC.

This paradoxical rise is largely attributable to the increasing prevalence of high-risk HPV infections, especially HPV16^[5]^. HPV is a circular double-stranded DNA virus that infects the oropharyngeal epithelium^[4]^. The unique anatomy of the oropharynx, particularly tonsillar crypts and the base of tongue, facilitates viral persistence and integration^[4]^. Changing sexual behaviors, particularly the increased prevalence of oral sex, have driven increasing HPV infection rates among younger individuals^[5]^.

Moreover, several diagnostic challenges persist. Currently, p16 immunohistochemistry is widely used as a surrogate marker for HPV-related OPSCC, yet this method can may yield false positives and does not directly assess transcriptionally active HPV. More specific assays, such as HPV DNA and RNA *in situ* hybridization or PCR-based detection, are recommended but remain underutilized^[4]^. Additionally, HPV + OPSCC is a biologically heterogeneous disease, with subgroups defined by age, smoking history, and tumor microenvironment composition exhibiting varied clinical outcomes^[5]^. For example, HPV + OPSCC patients with a significant tobacco exposure history (>10 pack-years) show worse prognosis despite HPV positivity. Developing robust biomarkers for risk stratification and personalized treatment selection remains an urgent clinical need.

## Molecular mechanisms and immune regulation

The oncogenesis of HPV + OPSCC is primarily driven by the viral oncoproteins E6 and E7. E6 promotes p53 degradation, impairing DNA repair and apoptotic responses, while E7 inactivates Rb, driving unchecked cell cycle progression^[4]^. This virus-mediated process bypasses the classic multi-step mutational pathways seen in tobacco-related head and neck cancers.

HPV also exerts sophisticated immune evasion strategies. The virus maintains low-level expression in basal epithelial layers and selectively expresses immunogenic proteins in differentiated layers, limiting immune detection. Additionally, HPV infection induces upregulation of PD-L1, suppression of TLR9, and may interfere with cytosolic DNA sensing pathways such as cGAS-STING, thereby attenuating innate immune responses^[1,6]^.

Emerging evidence also implicates extracellular vesicles in immune modulation. HPV + HNSCC-derived exosomes carrying miR-9 can promote M1 macrophage polarization, enhancing radiosensitivity^[7]^. Furthermore, the E7 protein degrades AMBRA1, inhibiting autophagy and sensitizing tumor cells to cisplatin-induced apoptosis^[8]^. Recent single-cell RNA sequencing studies have further elucidated the complex immune landscape of HPV + OPSCC, revealing enriched populations of activated CD8 + T cells, regulatory T cells, and exhausted T cells, as well as distinct macrophage subtypes^[9]^. These findings offer promising avenues for developing tailored immunotherapeutic strategies.

## Treatment strategies and progress

HPV + OPSCC patients generally exhibit enhanced sensitivity to radiation and chemotherapy and enjoy markedly improved long-term survival compared to HPV-negative counterparts^[4]^. Consequently, there is growing interest in treatment de-intensification, aiming to reduce toxicity while preserving excellent disease control.

The CheckMate 358 trial demonstrated that neoadjuvant PD-1 blockade with Nivolumab induced high pathological response rates and was well tolerated in resectable HPV + HNSCC^[10]^. Similarly, the ORATOR2 trial explored transoral surgery combined with low-dose radiotherapy and suggested improved quality of life, although oncologic outcomes require further confirmation^[11]^. Additional trials, such as ECOG 1308 and DAHANCA, are investigating risk-adapted de-escalation strategies tailored to tumor biology and patient characteristics^[4]^.

Nonetheless, treatment challenges remain. High-risk subgroups—including older patients, smokers, and those with large-volume nodal disease—still face elevated risks of recurrence and require careful selection of treatment intensity and vigilant post-treatment surveillance^[6]^. Furthermore, the optimal role and timing of immunotherapy in the definitive and adjuvant settings remains to be defined through future studies. Combining immunotherapy with standard chemoradiation or integrating it into de-escalation protocols represents a promising future direction, particularly for improving outcomes in high-risk HPV + OPSCC patients.

## Prevention strategies and future directions

HPV vaccination is the cornerstone of primary prevention for HPV-related cancers. The 9-valent HPV vaccine provides broad protection against oncogenic HPV types, including HPV16, and has demonstrated efficacy in preventing oral HPV infections^[4]^. Despite this, global vaccine uptake remains suboptimal, particularly in low- and middle-income countries, where access and cultural barriers persist^[3]^. In addition, male vaccination rates lag behind those of females in many regions, even though males bear a disproportionately high burden of HPV + OPSCC.

Moreover, effective screening tools for oropharyngeal precancerous lesions are currently lacking, limiting opportunities for early detection. ctHPV DNA assays show promise for non-invasive monitoring of treatment response and early detection of recurrence^[12]^. Dynamic changes in ctHPV DNA levels have been correlated with therapeutic outcomes and disease recurrence, suggesting potential utility in guiding post-treatment surveillance and personalized management^[4]^. Wider clinical implementation and prospective validation of ctHPV DNA assays should be prioritized in future trials.

## Conclusion and outlook

HPV + OPSCC represents a distinct clinical entity characterized by a well-defined viral etiology, unique molecular pathogenesis, and favorable treatment responses. Clinical management is evolving toward precision medicine, integrating molecular biomarkers, risk stratification, and individualized therapy. The review provides an excellent foundation for understanding the biology of HPV-driven cancers.

Looking ahead, future research should prioritize the identification of precancerous lesions, standardization of HPV detection methodologies, optimization of immunotherapy and combination regimens, and expansion of global vaccination efforts. Furthermore, strengthening international collaboration and harmonizing research efforts across diverse geographic regions will be critical to addressing the global burden of HPV + OPSCC. Through comprehensive, multidisciplinary approaches, we can advance the full-spectrum management of HPV + OPSCC and improve outcomes and quality of life for affected patients worldwide.

## Data Availability

No new data were generated or analyzed in this study.
